# Malaria parasite prevalence and Haematological parameters in HIV seropositive patients attending the regional hospital Limbe, Cameroon: a hospital-based cross-sectional study

**DOI:** 10.1186/s12879-019-4629-4

**Published:** 2019-11-21

**Authors:** Sorelle Mekachie Sandie, Irene Ule Ngole Sumbele, Martin Mih Tasah, Helen Kuokuo Kimbi

**Affiliations:** 1 Department of Zoology and Animal Physiology, University of Buea, Buea, Cameroon; 2 Department of Medical Laboratory Science, University of Bamenda, Bambili, Cameroon

**Keywords:** Malaria, Anaemia, Haematological parameters, HIV, Cameroon

## Abstract

**Background:**

Malaria and the human immunodeficiency virus (HIV) infection constitute public health problems in Cameroon including the South West Region (SWR). This study determined the prevalence of malaria parasites and haematological abnormalities in HIV positive patients in Limbe, Cameroon from April–July 2014.

**Methods:**

The study was cross-sectional and involved 411 participants who were administered structured questionnaires to record socio-demographic and clinical data. Three hundred and nine (309) HIV positive patients and one hundred and two (102) HIV negative individuals were examined clinically and venous blood collected for malaria parasite detection, HIV infection diagnosis and full blood count analysis.

**Results:**

Overall malaria parasite prevalence was 14.1% (58/411). This prevalence was significantly higher (*P* <  0.001) in the HIV negative participants (33.3%, 34/102) compared to the HIV positive patients (7.8%, 24/309). Amongst HIV positive participants, malaria parasite prevalence was significantly higher in female patients (*P* = 0.003), febrile patients (*P* <  0.001), anaemic patients (*P* = 0.015) and in patients who were not on antiretroviral treatment (ART) (*P* = 0.03) when compared with their respective counterparts. Among the HIV negative group, though not significant, malaria parasite prevalence was higher in females, febrile and anaemic patients when compared with their respective counterparts. Overall anaemia prevalence was 52.1% (214/309) and was significantly higher (*P* = 0.004) in HIV positive patients (56%, 173) than in HIV negative participants (40.2%, 41). Malaria/HIV co-infected patients had a significantly lower mean value of Hb (*P* = 0.002), RBC (P = 0.002) and Hct (*P* = 0.001) when compared with HIV-infected patients.

**Conclusion:**

HIV negative participants had a higher prevalence of malaria parasites than their HIV positive counterparts. Anaemia prevalence was higher in HIV positive patients than in HIV negative participants. Malaria/HIV co-infected patients presented with more red blood cell abnormalities than HIV-infected patients.

## Background

Malaria remains one of the most lethal human parasitic infections of our time with about 219 million cases and an estimated 435,000 malaria deaths recorded in 2017 [1]. Malaria is endemic in the 10 regions of Cameroon with an estimated prevalence of 29% [2]. The last decade has witnessed a massive scale up of malaria prevention efforts that have led to a significant reduction in malaria prevalence. HIV/AIDS infection further aggravates the morbidity in patients that are co-infected with malaria. The Cameroon government’s efforts to increase HIV infected patients’ access to anti-retroviral therapy (ART) is working, though not up to expectations. From 2012 to 2013, the treatment coverage rate ART increased from 20.5 to 26% [3]. ART slows the disease progression by preventing viral replication thereby decreasing the amount of virus in an HIV infected patient’s blood, that is, the viral load. In areas with stable malaria, HIV increases the risk of malaria infection and clinical malaria in adults, especially in those with advanced immunosuppression. Some ARTs are known to possess antimalarial properties, and some could act together with antimalarials against *P. falciparum* [4, 5]. Daily treatment with co-trimozaxole and ARTs have been associated with reduced prevalence of clinical malaria [6] and some protease inhibitors have specific antimalarial effects though the clinical relevance has not yet been established [7].

UNAIDS reported Sub-Saharan Africa in 2017 as the most severely affected region, with nearly 1 in every 25 adults (4.1%) living with HIV and accounting for nearly 2-thirds of the people living with HIV worldwide [8]. Reports from UNAIDS (2014) revealed that just 15 countries in the world (including Nigeria, South Africa, Uganda and Cameroon) account for more than 75% of the 2.1 million new HIV infections that occurred in 2015 [9]. According to the National AIDS Control Committee/Central Technical Group [3], there are 141 new HIV infections per day in Cameroon, which means six newly infected persons each hour, daily.

The National Institute of Statistics in collaboration with the Ministry of Public Health reported the South West Region as one of the regions with highest malaria and HIV prevalence; approximately 40 and 7.2%, respectively [3]. This is mostly due to the fact that the region is located in the tropical rain forest area and the majority of its population is made up of youths who are the most vulnerable group exposed to HIV. These two infections thus overlap in Southwest Cameroon and Limbe in particular [10]. The prevalence of *Plasmodium*/HIV co-infection in Cameroon varies from one region to the other; ranging from 2.24% in Bamenda [11] to 29.5% in Douala [12].

Both malaria and HIV lead to haematological abnormalities in the affected patients. The severity of haematological disease caused by *Plasmodium* especially *P. falciparum* is related to the ability of the parasites to invade and grow in different red cell populations as well as the intrinsic growth rate of the parasite [13]. Anaemia, leucopaenia and thrombocytopaenia are the most common haematological abnormalities resulting from both malaria and HIV infections especially in febrile patients [14, 15]. Leucocytosis has been associated with severe malaria [16] while leucopaenia is an abnormal condition commonly found in HIV patients [15]. The use of anti-retrovirals could positively or negatively affect these disorders. In both anti-retroviral-treated and untreated individuals, several types of haematological abnormalities are common [17]. Considering the enormous impact of these diseases on each other in the affected individuals, it is necessary to constantly generate epidemiological data on them in different settings in order to ascertain the likely changes in these parameters. The objectives of this study were to determine the prevalence of malaria parasites and malaria/HIV co-infections as well as to assess the variation in haematological parameters in malaria infected patients and HIV/malaria coinfected patients attending the Regional Hospital Limbe.

## Methods

### Study area

The study was carried out in the Regional Hospital Limbe (RHL), Fako Division, South West Region of Cameroon. Limbe is a coastal town situated at the foot of Mount Cameroon and opens the South West Region to the rest of the world through the Atlantic Ocean. It covers an area of 185 km^2^ and as of 2014 had a population of 84,500 inhabitants [18]. Its climate is typically equatorial with annual rainfall exceeding 4000 mm, temperatures ranging from 23 °C to 32 °C and 80% relative humidity as reported by the Cameroon Development Corporation. Limbe is characterised by two distinct seasons; a rainy season running from mid-March to October and a dry season running from November to mid-March.

### Study population

The study participants included HIV positive patients registered at the Voluntary Counselling Testing and Treatment Centre of the RHL and presumed HIV negative individuals presenting themselves at the Out Patient Department of the hospital for consultation during the study period. Participants were recruited irrespective of their age, sex, marital status, level of education, and with or without malaria-related signs and symptoms. Only those who gave their consent for blood collection and answered the questionnaire were enrolled in the study.

### Study design

This cross-sectional study was conducted from March–June, 2014. An information sheet and a brief talk were given to the participants, explaining the objectives and benefits of the study. Participants were then invited to participate in the study by signing an informed consent form. In the case of children (≤ 16 years) their parents signed a proxy consent form on their behalf. Before sample collection, a clinical examination was carried out followed by the administration of structured questionnaires to the participants. HIV-sero-negative individuals were persons at the hospital Out Patient Department who consented to be screened for HIV during the process and who tested negative for HIV.

The sample size was determined using the formula n = Z^2^pq/d^2^ [19] where n represented the sample size evaluated, Z was 1.96, which is the standard normal deviate (for the 95% confidence interval, CI), p was 58.9 and 51.1% [10], the malaria prevalence in HIV patients on ART and those not on ARV, respectively, q was 1-p and d was 0.05. The optimum sample size calculated was *n* = 380.

### Questionnaire

A pre-tested structured questionnaire was administered to all the consented participants enrolled in the study to obtain socio-demographic data such as age, sex, level of education, marital status; while clinical data such as HIV status, ART usage, duration on treatment were collected from each patient’s file at the HIV centre. The questionnaire also assessed the utilization of insecticide-treated nets and insecticide residual spray as preventive methods against malaria. An additional file shows the questionnaire used in detail (see Additional file 1).

### Laboratory procedure

Before blood sample collection, a participant’s axillary temperature was recorded using a digital thermometer. Fever was defined as temperature ≥ 37.5 °C. Under sterile conditions, a vacuum holder and vacutainer needle were used to collect 4 mL of venous blood into 2 well-labelled ethylenediaminetetraacetate (EDTA) tubes for each participant. A portion of the blood was used to prepare thick and thin blood films. The blood in one of the tubes was used for HIV rapid test (in the case of presumed HIV negative participants) and to conduct full blood count. Plasma was separated from the other portion of the blood, stored in an ice-filled cooler and transported to the Malaria Research Laboratory of the University of Buea for use in the HIV BISPOT assay. The prepared thin and thick blood films were Giemsa-stained and observed under the microscope following standard procedures [20] by two qualified microscopists. A complete blood count including values for white blood cell (WBC), red blood cell (RBC) and platelet counts, haemoglobin concentration (Hb), haematocrit (Hct), mean corpuscular volume (MCV), mean corpuscular haemoglobin (MCH), mean corpuscular haemoglobin concentration (MCHC), mean platelet volume (MPV), red cell distribution width (RDW), platelet distribution width (PDW), red blood cell distribution width coefficient of variation (RDW-CV), and red blood cell distribution width standard deviation (RDW-SD) was obtained using the URIT-3300 automated haematology analyser (URIT Medical Electronic CO., LTD. Jiuhua Road, Gungxi, China), following the manufacturer’s instructions. Anaemia was defined as Hb < 11.0 g/dL and further classified as described by Cheesbrough (2009) as severe (Hb <7 g/dl), moderate (Hb between 7.0 g/dl and 10.0 g/dl) and mild (>10.0 g/dl and <11 g/dl) [20, 21].

### HIV diagnosis

The Determine Rapid Test Kit (Abott Laboratories, CO., Ltd. Minato-Ku, Tokyo Japan) was used to test the supposed HIV negative subjects for HIV-1. Test results were read after 15 min from corresponding colour changes on the strip according to the manufacturer’s instructions.

All HIV positive results by the test kit were confirmed using the HIV-1 and 2 BISPOT assay (ORGENICS, Yavne, Israel). After bringing all components of the test kit and plasma samples to room temperature, 50 μL of each specimen and controls was dispensed into separate wells of row A of the developing plate and gently mixed. The comb was then introduced into the wells of row A and incubated for 10 min. The comb was later introduced into other wells serially with incubation and results were read by recognition of black dots on the comb teeth.

### Determination of CD4 T cell count

Ten (10) μL of Guava Auto CD4/CD4% antibody cocktail was placed into a 1.5 mL sample tube labelled with the patient’s serial number. A reverse pipette of 10 μL of whole blood (from the purple-top EDTA) was added onto the bottom of each tube containing the antibody cocktail. The tubes were vortexed immediately. The mixture was then incubated for 30 min at room temperature in the dark. Taking one tube at a time, 380 μL Guava 1X lysing solution was added into it and then the mixture vortexed immediately. The mixture was incubated again in the dark at room temperature for 15 min. Then each sample was acquired on a Guava PCA instrument with the Guava Auto CD4/CD4% software module and the CD4^+^ T-cell reading was recorded. CD4^+^ T-cell counts were categorized as low or advanced stage (< 200/μL), moderate or chronic stage (200–499/μL) and high or asymptomatic stage (≥500/μL) [12].

### Statistical analysis

The data obtained was Haematol using the IBM Statistical package for the Social Science (SPSS) version 20.0 software (IBM-SPSS Inc., Illinois, USA). Data was summarized into means and standard deviation (SD) and percentages were used in the evaluation of the descriptive statistics. The significance of differences in prevalence were explored using Pearson’s Chi-square or Fisher’s Exact test while Analysis of Variance (ANOVA), Kruskal-Wallis test or Student’s t test were used to assess difference in group means. Statistical significance was set at *P* <  0.05.

## Results

### Characteristics of the study population

A total of 411 participants (309 HIV positive patients and 102 HIV negative patients) with a median age of 37 (range 1–72) years participated in the study. The study population consisted of 299 (72.7%) females and 112 (27.3%) males distributed in 3 different age groups. The mean (± SD) ages of HIV positive and HIV negative subjects were 40.8 (± 10.2) years and 27.9 (± 15.7), respectively. The highest number of participants in the overall population was recorded in the 26–40 years age group (191, 46.5%). Only 8.3% (34) of the participants presented with fever. Amongst HIV positive patients, a total of 292 (94.5%) were under ART while 17 (5.5%) were not yet on ART, and 35.3% had CD4 T cell count ≥500 cells/μL of blood. The socio-economic, demographic and clinical data by HIV status of the study population is presented in Table 1.

**Table 1:** Baseline characteristics of the study population

Parameters	HIV POSITIVE	HIV NEGATIVE	TOTAL
	n (%)	n (%)	n (%)
Age groups (years)			
≤ 25	11 (3.6)	48 (47.1)	59 (14.4)
26–40	156 (50.5)	35 (34.3)	191 (46.5)
> 40	142 (46.0)	19 (18.6)	161 (39.2)
Sex			
Female	238 (77.0)	61 (59.8)	299 (72.7)
Male	71 (23.0)	41 (40.2)	112 (27.3)
Educational Level			
No formal	30 (9.7)	3 (3.4)	33 (8.3)
Primary	135 (43.7)	22 (25.0)	157 (39.5)
Secondary	124 (40.1)	45 (51.1)	169 (42.6)
Tertiary	20 (6.5)	18 (20.5)	38 (9.6)
ITN usage	196 (63.4)	53 (52.0)	249 (60.6)
IRS usage	78 (25.2)	26 (25.5)	104 (25.3)
Clinical			
CD4 T-cell count(cells/μl)			
< 200	41 (23.7)		41 (23.7)
200–499	71 (41.0)		71 (41.0)
> 500	61 (35.3)		61 (35.3)
ART usage	292 (94.5)		292 (94.5)
ART Duration (months)			
< 12	33 (10.8)		33 (10.8)
12–35	82 (26.8)		82 (26.8)
≥ 36	191 (61.8)		191 (61.8)
Fever prevalence	16 (5.2)	18 (17.6)	34 (8.3)
Malaria prevalence	24 (7.8)	34 (33.3)	58 (14.1)
Anaemia prevalence	173 (56.0)	41 (40.2)	214 (52.1)
Anaemia severity			
Mild	95 (54.9)	16 (39)	111 (51.9)
Moderate	69 (39.9)	22 (53.7)	91 (42.5)
Severe	9 (5.2)	3 (7.3)	12 (5.6)

### Malaria parasite prevalence

The overall prevalence of malaria in the study population was 14.1% (58). Malaria parasite prevalence was significantly higher (χ^2^ = 41.36, *P* <  0.001) in HIV negative (33.3%, 34) than HIV positive participants (7.8%, 24). The highest malaria parasite prevalence was recorded in the youngest age group in both HIV positive (36.4%, 4) and HIV negative (41.7%, 20) participants. As shown in Table 2, female participants among both HIV positive and HIV negative participants had a higher malaria parasite prevalence (8%, 19 in HIV positive and 34.4%, 31 in HIV negative) than their male counterparts (7% in HIV positive and 31.7% in HIV negative); although only statistically significant in HIV positive patients (χ^2^ = 11.98, *P* = 0.003). Malaria parasite prevalence was significantly higher (χ^2^ = 20.82, *P* = 0.001) in HIV positive patients who presented with fever (37.5%) than those who did not (6.1%). The same trend of malaria parasite prevalence was observed amongst HIV negative participants, although the difference was not statistically significant (χ^2^ = 1.29, *P* = 0.20). Anaemic patients had a higher prevalence of malaria parasite (11% in HIV positive and 39% in HIV negative) than non-anaemic patients (3.7% in HIV positive and 29.5% in HIV negative). This difference was statistically significant only among HIV positive patients (χ^2^ = 5.67, *P* = 0.01) as shown in Table 2. Out of the 214 anaemic patients, those with moderate anaemia had the statistically significant (χ^2^ = 7.44, *P* = 0.024) highest prevalence of malaria parasite (24.2%, 22). With respect to HIV status, analysis revealed that participants who had moderate anaemia (18.8% in HIV positive and 40.9% in HIV negative) had the highest prevalence of malaria parasite as compare to their respective counterparts; though the difference was only significant for HIV positive patients (χ^2^ = 7.53, *P* = 0.02**)** (Table 2).

**Table 2:** Malaria parasite prevalence in HIV positive and negative participants

Categories	HIV Positive			HIV Negative			Overall		
	n	Malaria parasite positive %(n)	χ^2^, P	n	Malaria parasite positive %(n)	χ^2^,P	N	Malaria parasite positive %(n)	χ^2^,P
Gender									
Female	238	8.0(19)	11.98	61	34.4(21)	0.068	299	13.4(40)	0.48
Male	71	7.0(5)	0.003^*^	41	31.7(13)	0.51	112	16.1(18)	0.29
Age (years)									
≤ 25	11	36.4(4)	13.06	48	41.7(20)	3.14	59	40.7(24)	40.34
26–40	156	6.4(10)	0.001	35	28.6(10)	0.21	191	10.5(20)	< 0.001^*^
> 40	142	7.0(10)		19	21.1(4)		161	8.7(14)	
Fever status									
Febrile	16	35(6)	20.82	18	44.4(8)	1.29	34	41.2(14)	22.40
Afebrile	293	6.1(18)	0.001^*^	84	31.0(26)	0.20	377	11.7(44)	< 0.001^*^
Anaemic status									
Anaemic	173	11.0(19)	5.67	41	39.0(16)	0.99	214	16.4(35)	1.854
Non-anaemic	136	3.7(5)	0.01^*^	61	29.5(18)	0.22	197	11.7(23)	0.11
Anaemia severity									
Mild	95	5.3 (5)	7.537	16	37.5 (6)	0.089	111	9.9 (11)	7.443
Moderate	69	18.8 (13)	0.02^*^	22	40.9 (9)	0.95	91	24.2 (91)	0.024^*^
Severe	9	11.1 (1)		3	33.3 (1)		12	16.7 (2)	
Educational level									
Primary	135	6.7(9)	5.78	22	40.9(9)	3.94	157	11.5(18)	5.68
Secondary	124	5.6(7)	0.05	45	22.2(10)	0.27	169	10.1(17)	0.20
Tertiary	20	15.0(3)		18	33.3(6)		38	23.7(9)	
None	30	16.7(5)		3	0.0(0)		33	15.2(5)	
ART use									
Yes	292	6.8(20)	6.23				292	6.8(20)	6.23
No	17	23.5(4)	0.03^*^				17	23.5(4)	0.03^*^
Duration on ART									
< 12 months	33	18.2(6)	6.05				33	18.2(6)	6.05
12–35 months	82	6.1(5)	0.06				82	6.1(5)	0.06
≥ 36 months	191	6.3(12)					191	6.3(12)	
CD4T-cell (cells/μl)									
< 200	41	4.9(2)	0.21				41	4.9(2)	0.21
200–499	71	7.0(5)	0.90				71	7.0(5)	0.90
≥ 500	61	6.6(4)					61	6.6(4)	

* Statistically significant *P* value

Among the 17 HIV positive participants who had not started the ART, prevalence of malaria parasite was higher (23.5%, 4) compared with the 292 patients under ARV therapy (6.8%, 20). This difference was statistically significant (χ^2^ = 6.23, *P* = 0.03). With respect to duration on ART, HIV positive participants who have been on therapy for ≤12 months had the highest prevalence of malaria parasite (18.2%, 6) compared with those who had been taking their medications for a longer period. However, the difference was not statistically significant (χ^2^ = 6.05, *P* = 0.06).

### Malaria preventive measures

In the overall population, participants not using insecticide treated nets (ITNs) had a higher prevalence of malaria parasites (16%, 26) than those who used ITNs (12.9%, 32), but the difference was not significant (χ^2^ = 0.57, *P* = 0.27). Participants using IRS as their preventive measure had a non-statistically higher prevalence of malaria parasites (16.3%, 17) than those not using it (13.4%, 41).

Among HIV positive participants, those who did not use ITN nor IRS had a higher malaria parasite prevalence (10.6% [12/113]), 8.2% [19/231]) than those who used ITN or IRS (6.1% [12/196], 6.4% [5/78]), respectively. However, the differences were not significant (*P* = 0.41, *P* = 0.12). Among HIV negative participants, those who did not use ITN nor IRS had a lower malaria parasite prevalence (28.6%, 14; 28.9%, 22 respectively) as compared to their respective counterparts (37.7%, 20; 46.2%, 12). These differences were not statistically significant (*P* = 0.8, *P* = 0.15).

**Fig. 1:**
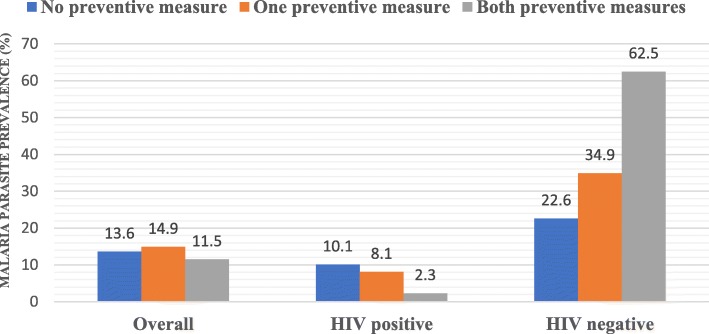
Malaria parasite prevalence with respect to implementation of malaria preventive measures

As shown in Fig. 1, when the two preventive methods were compared, the lowest malaria parasite prevalence was recorded among participants who used both preventive measures. When comparing the usage of both preventive measures among HIV positive and HIV negative participants, it was observed that those who did not implement any preventive measure had the highest malaria parasite prevalence in HIV positive patients (10.1%, 8/79) which was the opposite among HIV negative participants; those not implementing preventive measures had the lowest malaria parasite prevalence (22.6%, 7/31) (Fig. 1).

### Anaemia prevalence and Anaemia severity

The overall prevalence of anaemia in the study population was 52.1% (214). Anaemia prevalence was significantly higher (χ^2^ = 7.00, *P* = 0.004) in HIV positive patients (56%, 173) than their HIV negative counterparts (40.2%, 41). HIV positive patients and HIV negative participants in the age groups 26–40 years (59.0%) and ≤ 25 years (52.1%), respectively had the highest prevalence of anaemia as compared to their age-group counterparts, though the differences were not statistically significant. Anaemia prevalence was higher in female participants (64.7%, 154 in HIV positive and 49.2%, 30 in HIV negative) compared to male participants (26.8%, 19 in HIV positive and 26.8%, 11 in HIV negative). These differences were statistically significant for both HIV positive patients (*P* = 0.001) and their HIV negative counterparts (*P* = 0.02). With respect to malaria status, both in HIV positive and HIV negative participants, anaemia prevalence was higher in those who were positive for malaria parasite than those who were not. Within both categories of patients, febrile patients had a higher prevalence of anaemia (56.2%, 9 in HIV positive and 55.6%, 10 in HIV negative) than their afebrile counterparts (56%, 164 in HIV positive and 36.9%, 31 in HIV negative). HIV positive patients with CD4 T-cell count < 200 cells had the highest prevalence of anaemia (73.2%) that approached significance (*P* = 0.05) when compared to their counterparts. As shown in Table 3, although not significant (*P* = 0.31), HIV patients not on ART had a higher prevalence of anaemia (64.7%, 11) than those on ART (55.5%, 162).

**Table 3:** Prevalence of anaemia as affected by sociodemographic and clinical factors in HIV positive and HIV negative participants

Categories	HIV Positive			HIV Negative			Overall		
	n	Anaemia prevalence (n)	χ^2^ P	n	Anaemia prevalence (n)	χ^2^ P	N	Anaemia prevalence (n)	χ^2^ P
Gender									
Female	238	64.7(154)	31.9	61	49.2(30)	5.16	299	61.5(184)	39.4
Male	71	26.8(19)	0.001^*^	41	26.8(11)	0.02^*^	112	26.8(3 + 0)	< 0.001^*^
Age group (years)									
≤ 25	11	54.5(6)	1.15	48	52.1(25)	5.37	59	52.5(31)	0.34
26–40	156	59.0(92)	0.56	35	28.6(10)	0.07	191	53.4(102)	0.84
> 40	142	52.8(75)		19	31.6(6)		161	50.3(81)	
Malaria status									
Malaria positive	24	79.2(19)	5.67	34	47.1(16)	0.99	58	60.3(35)	1.85
Malaria negative	285	54.0(154)	0.01	68	36.9(25)	0.22	353	50.7(79)	0.11
Fever status									
Febrile	16	56.2(9)	0.001	18	55.6(10)	χ^2^ = 2.14	34	55.9(19)	0.21
Afebrile	293	56.0(164)	0.50	84	36.9(31)	*P* = 0.11	377	51.7(195)	0.38
ARV use									
Yes	292	55.5(162)	0.55				292	55.5(162)	0.55
No	17	64.7(11)	0.31				17	64.7(11)	0.31
Duration on ARV									
< 12 months	33	63.6(21)	0.86				33	63.6(21)	0.86
12–35 months	82	56.1(46)	0.42				82	56.1(46)	0.42
≥ 36 months	191	55.0(105)					191	55.0(105)	
CD4T-cell (cells/μl)									
< 200	41	73.2(30)	3.78				41	73.2(30)	3.78
200–499	71	60.6(43)	0.05				71	60.6(43)	0.05
≥ 500	61	54.1(33)					61	54.1(33)	

* statistically significant *P* value

With respect to anaemia severity, 51.9% (111) of the anaemic patients had mild anaemia, 42.5% (91) had moderate anaemia and 5.9% (12) had severe anaemia in the overall study population.

**Fig. 2:**
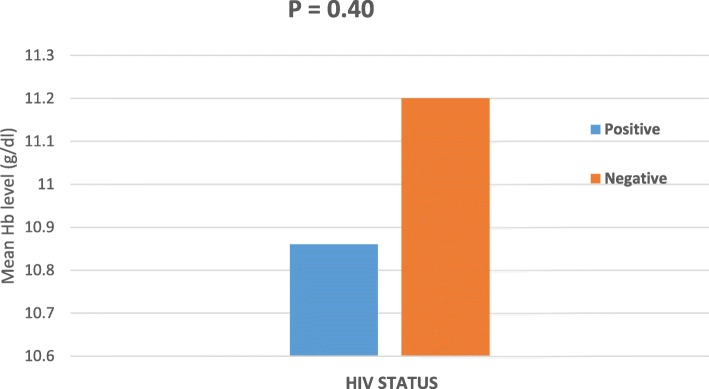
Mean Haemoglobin level (g/dl) with respect to HIV status

As shown in Fig. 2, HIV positive patients had lower mean haemoglobin level when compared to HIV negative participants; though the difference in mean was not statistically significant (*P* = 0.40).

### Effect of malaria/HIV co-infection on Haematological parameters

Malaria/HIV co-infection was observed in 24 patients (5.8%) while those who had only malaria were 34 (8.3%). The mean values of 14 haematological parameters were compared between malaria/HIV co-infected patients and only HIV-infected patients. Seven haematological parameters (Hb, RBC, MCV, MCH, Lymphocyte count, Hct, PDW) showed lower mean value among malaria/HIV co-infected patients when compared with HIV-infected patients. The difference was statistically significant for Hb (*P* = 0.002), RBC (P = 0.002) and Hct (*P* = 0.001). Conversely, co-infected patients were observed to have higher mean values for WBC, PLT, MCHC, Granulocyte count, MPV, RDW-SD and RDW-CV than HIV-infected patients without malaria. The difference was not statistically significant as shown in Table 4.

**Table 4:** Comparison of haematological parameters between HIV positive (*n* = 285) and HIV/malaria co-infected patients (*n* = 24) among the study population

Haematological Parameters	Malaria/HIV co-infected (24)	HIV-infected (285)	T-test	*P*-values*
Hb g/dl	9.75 ± 1.67	10.95 ± 1.87	3.331	0.002*
RBC × 10^12^/l	3.32 ± 0.53	3.73 ± 0.66	3.442	0.002*
WBC ×10^9^/l	6.12 ± 3.88	5.88 ± 2.51	−0.301	0.761
PLT ×10^9^/l	170.96 ± 79.01	168.58 ± 65.03	−0.147	0.890
MCV fl	94.63 ± 9.97	94.99 ± 10.37	0.173	0.862
MCHC g/dl	31.34 ± 1.04	31.19 ± 3.26	−0.526	0.601
MCH Pg	29.60 ± 3.26	29.61 ± 4.63	0.015	0.984
Lymphocyte Diff %	26.82 ± 15.38	29.81 ± 11.38	0.933	0.362
Granulocyte Diff %	62.33 ± 14.47	58.35 ± 11.86	−1.311	0.20
Hct %	30.8 ± 5.43	35.26 ± 6.06	3.814	0.001*
RDW_CV fl	13.54 ± 2.51	13.38 ± 5.13	−0.265	0.797
RDW_SD %	51.54 ± 8.15	50.15 ± 5.68	−0.823	0.422
MPV fl	11.55 ± 1.67	11.43 ± 1.76	−0.326	0.753
PDW	8.46 ± 1.62	8.99 ± 4.55	1.247	0.221

Values are mean ± SD

*Statistically significant P value

*P* values were calculated using independent student t-test

## Discussion

This study was a hospital-based cross-sectional study carried out to determine the prevalence of malaria parasites in HIV positive and HIV negative individuals at the Limbe Regional Hospital. Irrespective of the participants’ HIV status, the overall malaria parasites prevalence was 14.1%. This result is lower than the reported national malaria prevalence of 29% [22] and also lower than the 29.4% recorded in Douala [23] and 55.0% recorded in Limbe [10]. This decrease could be as a result of the sustained malaria control strategies put in place by the Cameroon government over the past years both in HIV positive and HIV negative population. These strategies include the sensitization campaigns on how to better use LLINs, free distribution of ART to patients together with cotrimoxazole, increased number of care and treatment centers for HIV/AIDS patients and the use of Artemisinin-combination therapy (ACT) as recommended by World Health Organization.

Interestingly, the prevalence of malaria parasites was higher in HIV negative participants (33.3%) than their HIV positive counterparts (7.8%). This is in line with a study carried out in Ethiopia [24] which also reported a higher malaria parasites prevalence in HIV negative participants than in their HIV positive counterparts. HIV positive patients attending HIV specialty unit are more likely to receive routine medical care follow-up and health maintenance compared to patients at the Outpatient department The fact that HIV negative participants were recruited from the Outpatient department of the hospital could also contribute to this high malaria parasites prevalence among them; as majority came to the hospital to seek for medical care due to the presence of acute symptoms of different illness including malaria. This finding also correlates with that of Njunda et al. [11] in Bamenda which reported a low prevalence of malaria parasites in HIV positive patients.

In accordance with Kasirye et al. [25], Amuta et al. [26] and Gennano et al. [27] malaria parasites prevalence was higher in HIV positive patients who were not on ART than those on ART. This may be likely due to the reconstitution of their immune system associated with the administration of the drugs, indicating that the ART is effective. The low prevalence in patients on ART could be as a result of the presence of cotrimoxazole as part of their therapy which has been shown to have some antimalarial properties that reduce the incidence of malaria in HIV patients. WHO recommends to stop cotrimoxazole in clinically stable patients with evidence of viral suppression under ART, while it should be continued in patients living in areas with high malaria and bacterial infection prevalence [28]. That is the case with Cameroon where cotrimoxazole is continuously given to all HIV patients irrespective of their viral suppression levels.

Findings from the study also revealed that in both HIV positive and HIV negative patients, those who presented with fever had significantly higher malaria parasite prevalence than those who did not have fever. This confirms that the fever observed was likely because of the malaria parasite infection in the patient and this is in line with a study carried out in Gabon by Bouyou-Akotet et al. [29].

In this study, participants aged ≤25 years of age were more infected with malaria parasites than participants of other age groups in both study groups. This result could have been due to the fact that the age group ≤25 years includes children who are a vulnerable group when it comes to malaria as their immune system is still developing and also the young adults who stay out late thereby increasing chances to be bitten by an infected mosquito. This finding is in line with results reported form other studies carried out in the Southwest region of Cameroon by Apinjoh et al. [30]; Ebai et al. [31]; Amuta et al. 2012 [26] and I et al. [32].

Females had a higher prevalence of malaria parasites as compared to the males in this study. The fact that more females were recruited for this study more than males could have accounted for the difference as majority of males avoid coming for check-up in the hospital as a result of stigmatization [33]. This result is in line with a study carried out by Kimbi et al., [34] and contrary to what has been reported in other studies [35, 36] where males had a higher prevalence of malaria parasite due to the fact that they often engage themselves in late night activities and also often expose their chest, especially when the weather is hot and during farm work.

From this study, it was observed that anaemic patients had a higher prevalence of malaria parasites when compared with those who were not anaemic in both study populations. Malaria parasites cause destruction of red blood cells hence reducing haemoglobin levels leading to anaemia. This relationship has been well established in several studies in Cameroon and Africa [21, 37, 38].

Findings from the study revealed that a suitable number of participants used ITNs in their homes. The prevalence of malaria parasites was lower in those who used ITNs when compared with those who did not. This is because ITNs serves as a barrier to mosquitoes and so preventing transmission of the malaria parasites. This result is in line with findings in Kenya [39] which reported that sleeping under an ITN considerably reduces the risk of man-vector contact and consequently malaria infection. Among HIV positive patients, those who implemented two different preventive measures against malaria had the lowest malaria parasite prevalence. This is an indication that combination different preventive measures against malaria helps to better combat this infection. This finding give credit to the efficiency of possible integrated vector control measures against malaria. This aspect needs to be encouraged among both HIV positive and HIV negative individuals and emphasis made to HIV positive patients during their daily lectures at the HIV Centre.

In this study, the prevalence of anaemia was significantly higher in HIV positive patients than HIV negative participants. Several drugs used to combat HIV and its complications must have contributed to this high anaemia prevalence. Zidovudine has been reported to cause anaemia because of myelo-suppression [40]. Anaemia is also caused by different etiological agents including HIV-associated malignancies [41]. This result is in line with that reported by Ayukenchengamba et al. [42] and Ojurongbe et al. [43] in other studies.

In both HIV positive and HIV negative populations, females had a significantly higher prevalence of anaemia than males. This difference could be attributed to the menstrual blood loss in women and to the drains on iron stores that occur with pregnancy and delivery [41].

From our study, it was observed that mean value of Hb, RBC and HCT were significantly lower in malaria/HIV co-infected patients as compared with HIV-infected patients. This finding is consistent with previous studies done in Nigeria [44, 45] and Southeast Ethiopia [46]. This can be attributed to the combined effects of RBC destruction caused by the malaria parasites and the administration of Zidovudine which has been reported to cause anaemia.

This study also found that the mean lymphocyte count, granulocyte count and WBC were similar between HIV-infected patients and malaria/HIV co-infected patients (*P* > 0.05). This observation agrees with previous findings by Erhador et al. [44] and Tchinda et al. [15].

## Conclusion

In general, malaria parasite prevalence was higher in HIV negative than in HIV positive participants attending the Regional Hospital Limbe. The prevalence of anaemia was high in the overall population and was the main haematological abnormality observed amongst HIV positive patients. Three blood parameters (Hb, RBC, Hct) were significantly different between malaria/HIV co-infected patients and HIV-infected patients. These findings also revealed that malaria may not be the primary cause of haematological abnormalities in these patients. To confirm these results, a community-based cross-sectional study needs to be carried out to have a clear picture of the malaria parasite prevalence of HIV negative subjects in the community; which could be later on compared with their HIV positive counterparts. The study had as limitation the fact that both categories of participants visited the hospital for different reasons; one to seek for medical attention at the OPD while the other for normal routine follow-up. So these different reasons to some extend could have contributed to the difference in malaria prevalence and other clinical parameters. Therefore, prospective studies with large sample size from other settings are needed to substantiate these findings.

## Supplementary information

**Additional file 1.** Questionnaire, Socio-demographic and clinical data of the participants.

## Data Availability

All datasets on which the conclusions of the research rely are presented in this paper. However, data is available from the corresponding author on reasonable request.

## Abbreviations

ACT: Artemisinin Combination Therapy
AIDS: Acquired Immune Deficiency Syndrome
ANOVA: Analysis of Variance
ART: Anti-retroviral Therapy
ARV: Anti-retroviral (drug)
CD4 T: T-lymphocyte cell bearing CD4 receptors
CDC: Center for Disease Control and Prevention
CNLS: Comité National de Lutte contre le SIDA
GTC: Groupe Technique Central National
Hb: Haemoglobin
HIV: Human Immunodeficiency Virus
IRS: Insecticide Residual Spraying
ITNs: Insecticide-Treated Nets
MCH: Mean Corpuscular Haemoglobin
MCHC: Mean Corpuscular Haemoglobin Concentration
MCV: Mean Corpuscular Volume
MPV: Mean Platelet Volume
OIHC: Office for Institutional HIV Co-ordination
OPD: Out Patient Department
P: Probability Level
PLT: Platelets
RBCs: Red Blood Cells
RDW: Red Cell Distribution Width
RHL: Regional Hospital Limbe
SPSS: Software Package of Social Sciences
UNAIDS: Joint United Nations Programme on HIV/AIDS
WBC: White Blood Cell
WHO: World Health Organization
