# Supplementary material for: Malaria parasite prevalence and Haematological parameters in HIV seropositive patients attending the regional hospital Limbe, Cameroon: a hospital-based cross-sectional study
Source: BMC Infect Dis. 2019 Nov 21;19:988. doi: 10.1186/s12879-019-4629-4 (PMC6873725; doi:10.1186/s12879-019-4629-4)
Supplement: Supplementary file 1 — Additional file 1. Questionnaire, Socio-demographic and clinical data of the participants. [file 12879_2019_4629_MOESM1_ESM.docx]

**QUESTIONNAIRE FOR SUBJECTS**

**Study Title:** Malaria Parasite Prevalence and Haematological Parameters in HIV Seropositive Patients Attending The Regional Hospital Limbe, Cameroon: A Hospital-Based Cross-Sectional Study

Patient’s identification code: ____________ Date of enrolment: ________________

**Socio-demographic data**

Sex: M F Age: ______

Occupation: __________________________

Quarter of residence: ___________________

Address/contact: _______________________

Marital status: Married Divorced Single Widow(er)

Level of education: No formal Primary Secondary Tertiary

**Clinical evaluation:**

Temperature ________ oC

HIV status: _____________

Malaria Test result: ________________

Are you taking antiretroviral therapy? Yes No

If yes, which year did you start taking them? _____________

CD4 T cell count: _____________

**Malaria Prevention methods used**

1. Do you use insecticide Residual spray (IRS) to kill mosquitoes? Yes No
2. Do you sleep under a mosquito net? Yes No
